# Investigating the role of HMGA2 plasma level as a diagnostic marker in bladder urothelial carcinoma patients

**DOI:** 10.1007/s00432-025-06192-z

**Published:** 2025-04-09

**Authors:** Farah Khazem, Almoutassem Billah Zetoune

**Affiliations:** Department of Biochemistry and Microbiology, Faculty of Pharmacy, Damascus University, Damascus, Syria

**Keywords:** Bladder cancer, Urothelial carcinoma, HMGA2, Plasma, BC stage, BC grade, NMIBC, MIBC, Pyroptosis

## Abstract

**Background:**

Bladder Cancer (BC) is an environmental cancer caused by exposure to a globally widespread carcinogen, which is smoking, and it is characterized by high rates of recurrence and mortality. High Mobility Group A2 (HMGA2) protein is an oncofetal protein that belongs to the HMG family proteins. It is involved in various stages of carcinogenesis and cancer progression. This study investigated the presence and levels of the HMGA2 protein in bladder urothelial carcinoma patients’ plasma and in healthy individuals and their association with the clinicopathological features of bladder urothelial carcinoma.

**Methods:**

This case–control study included 80 individuals divided into two groups: a healthy group (*n* = 22) and a patient group with bladder urothelial carcinoma (*n* = 58). There were 16 patients with Muscle-Invasive Bladder Cancer (MIBC) and 42 patients with Non-Invasive Bladder Cancer (NMIBC) in the patients’ cohort according to the European Association of Urology (EAU) classification. HMGA2 plasma levels were measured by Sandwich Enzyme-Linked ImmunoSorbent Assay (ELISA). The statistical analysis was performed using IBM SPSS statistics (version 25) software. The *t*-test and the Mann–Whitney test were used.

**Results:**

Plasma HMGA2 protein levels were higher in the BC group than in the healthy group (*P* < 0.001), they also were higher in MIBC (pT2-pT3) than in NMIBC (pTa-pT1) (*P* < 0.001). HMGA2 plasma levels were higher in high grade BC patients than in low grade BC patients (*P* = 0.049).

**Conclusions:**

This study confirmed that the plasma HMGA2 protein level was higher in bladder cancer patients than in healthy individuals and that its elevated plasma levels were correlated with advanced stage and grade of BC; thus, the plasma HMGA2 protein level represents a potential non-invasive marker that could be included in bladder cancer diagnosis approach.

## Background

Bladder Cancer (BC) represents approximately 3.1% of all newly diagnosed cancer cases and 2.3% of all deaths resulting from cancer globally according to the Global Cancer Observatory (GLOBOCAN) statistics of 2022, and in Syria in particular there were 721 new cases and 396 deaths due to it (WHO 2024). Urothelial carcinoma is the most common histological type of BC. BC is considered an occupational and environmental cancer due to its occurrence as a result of continuous exposure to environmental factors that are carcinogenic to the urinary epithelium, and the chief factor is smoking, which is responsible for 50% of BC cases (Wong et al. 2021), in addition to exposure to aromatic amines, polycyclic aromatic hydrocarbons, and chlorinated hydrocarbons, which are commonly found in industrialized areas (Nabavizadeh et al. 2021; Mushtaq et al. 2019). BC is characterized by a high risk of recurrence and mortality (Yao et al. 2022) because of its histological and molecular characteristics and delayed diagnosis, as hematuria is the main symptom and appears in only 70% of patients (Degeorge et al. 2017). The presence of the tumor is then confirmed by cystoscopy and other invasive procedures that can lead to many complications and discomforts to the patient (Wong et al. 2021). The ideal non-invasive marker has not yet been approved.

Many studies have indicated that the High Mobility Group A2 (HMGA2) protein is a potential diagnostic and prognostic cancer marker due to its multifaceted role in cancer development and progression (Ding et al. 2014; Wu et al. 2016; Mansoori et al. 2020, 2021a; Gao et al. 2017; Mahajan et al. 2010; Hengjuan Lv et al. 2019; Jin et al. 2011; Yang et al. 2011). The HMGA2 protein is an oncofetal protein that belongs to the HMG family proteins and the HMGA subfamily. These proteins are a distinct category of transcription factors designated of “architectural factors” due to their lack of direct transcriptional activity (Asher et al. 1995). The HMGA2 protein exhibits a high degree of plasticity attributed to its intrinsically disordered structure, which is a distinct feature of HMGA proteins. This structural flexibility is hypothesized to enable HMGA proteins to interact with DNA, modify its conformational state and engage with a diverse array of proteins including numerous transcription factors, causing the modulation of the three-dimensional structure of chromatin by binding to AT-rich regions in the minor grooves of DNA through their AT hooks, thereby regulating the expression of numerous genes involved in carcinogenesis and cancer progression (Vignali and Marracci 2020; Reeves 2003; Thi-Hai Pham et al. 2020). Many studies have reported the elevated levels of the HMGA2 protein in cancer tissues and correlated its expression levels with clinicopathological features in various types of cancer, including bladder (Ding et al. 2014; Yang et al. 2011), breast (Mansoori et al. 2020, 2021a), colon (Wang et al. 2022), lung (Gao et al. 2017), ovarian (Mahajan et al. 2010), and liver cancers (Hengjuan Lv et al. 2019).

These findings, in addition to the detection of HMGA2 protein in few body fluids such as pancreatic cyst fluid and liver cancer patients’ serum (DiMaio et al. 2016; Huang et al. 2022), prompted us to investigate the presence and levels of the HMGA2 protein in BC patients’ plasma, as no data are available concerning the detection of the HMGA2 protein in the plasma of BC patients, and there is a vital need to identify a marker that supports the procedures used to diagnose bladder cancer.

## Materials and methods

### Patients and study design

This case–control study included 80 individuals divided into two groups: a healthy group (*n* = 22) and a BC patient group with urothelial carcinoma (*n* = 58). There were 16 patients with Muscle-Invasive Bladder Cancer (MIBC) and 42 patients with Non-Invasive Bladder Cancer (NMIBC) in the BC patient cohort according to the European Association of Urology (EAU) classification depending on the depth of the muscle invasion.

Patients included in this study were newly diagnosed bladder urothelial carcinoma patients with no history or current incidence of other tumors, and no treatment was initiated. On the other hand, this study excluded patients with creatinine levels higher than normal (to eliminate the effect of renal dysfunction on protein clearance, due to its relatively small size), patients who were diagnosed with any necrotizing injury (to deny any cause–other than bladder cancer–increases the release of the HMGA2 protein from the cells), and patients diagnosed with benign tumors of any type. While healthy individuals were selected based on the absence of any clinical symptoms or laboratory evidence indicating the presence of an inflammatory condition.

### HMGA2 assay

Blood samples were drawn from patients attending the Urology Department at The National University Hospital just before underwent TransUrethral Resection of Bladder Tumor (TURBT) between January 2023 and October 2023. Patient information was obtained by personally interviewing and reviewing patient medical files, while clinicopathological data were obtained from the hospital pathology laboratory**.**

HMGA2 plasma levels were measured by Sandwich Enzyme-Linked ImmunoSorbent Assay (ELISA) using a commercial human HMGA2 ELISA kit (ELK Biotechnology, China). ELISA assay was performed according to the manufacturer’s instructions.

### Ethical approval

This study obtained approval from the Biomedical Research Ethics Board (BMREC) at Damascus University on (2022/1/29) (ID number: PH-290122 -31), and blood samples were drawn from the participants after they provided written informed consent.

### Statistical analysis

The statistical analysis was performed using IBM SPSS statistics (version 25) software (SPSS Inc., Chicago, IL, USA). Statistical differences between normally distributed values were tested using *t* test, while the differences between abnormally distributed values were tested using Mann–Whitney tests. All normally distributed values were presented as Mean ± Standard deviation (Std.), while abnormally distributed values were presented as Median [Interquartile Rang] [IQR]. A *P* value < 0.05 was considered to indicate statistical significance.

## Results

### Demographic data of the study population

This study included 80 individuals divided into two groups: a healthy group (*n* = 22) and a BC patient with urothelial carcinoma group (*n* = 58). The mean age of the BC patient group was (60.52 ± 10.94 years), while that of the healthy control group was (55.29 ± 7.47 years). Table 1 shows the distribution of the study population according to gender, smoking status, and clinicopathological features.

**Table 1:** The distribution of the study individuals according to gender, smoking status, and clinicopathological features

Parameter	Case, *n* (%)	Plasma HMGA2 level (ng/ml)	*P*-value
Gender			
Healthy group			
Male	14 (63.64%)	6.27 ± 1.99	0.630
Female	8 (36.36%)	5.90 ± 0.93	
Gender			
BC patient group			
Male	50 (86.21%)	12.67 [10.17–15.57]	0.718
Female	8 (13.79%)	12.52 [11.92–16.18]	
Smoking status			
Healthy group			
Smoker	5 (22.73%)	5.3 ± 1.08	0.204
Non-smoker	17 (77.27%)	6.4 ± 1.74	
Smoking status			
BC patient group			
Smoker	44 (75.86%)	12.87 [10.05–15.51]	0.757
Non-smoker	14 (24.14%)	12.29 [11.01–15.74]	
Study groups			
Healthy individuals	22	6.48 [4.95–7.01]	< 0.001*
BC patients	58	12.67 [10.28–15.57]	
BC stage			
pTa	7 (12.07%)	11.04 ± 3.02	–
pT1	35 (60.34%)	11.73 ± 2.49	–
pT2	5 (8.62%)	15.26 ± 1.50	–
pT3	11 (18.97%)	18.70 ± 3.47	–
pTa vs. pT1	–	11.04 ± 3.02 vs 11.73 ± 2.49	1
pT2 vs. pT3	–	15.26 ± 1.50 vs 18.70 ± 3.47	0.117
(pTa-pT1) vs. (pT2-pT3)	–	11.62 ± 2.56 vs 17.62 ± 3.37	< 0.001*
Muscle invasion			
NMIBC	42 (72.41%)	11.62 ± 2.56	< 0.001*
MIBC	16 (27.59%)	17.62 ± 3.37	
BC grade			
Low grade	22 (37.93%)	10.63 [8.6–13.68]	0.049*
High grade	36 (62.07%)	13.34 [11.7–17.28]	

*BC* bladder cancer; *MIBC* muscle-invasive bladder cancer; *NMIBC* non-invasive bladder cancer

Values with normal distribution were presented as (Mean ± Std.) and groups were compared using the t test, while those with abnormal distribution were presented as (Median [IQR]) and groups were compared using Mann–Whitney test. Values with a significant statistical difference (*P*-value < 0.05) were indicated by (*).

### Comparison of plasma HMGA2 levels between the BC group and healthy group

**Fig. 1:**
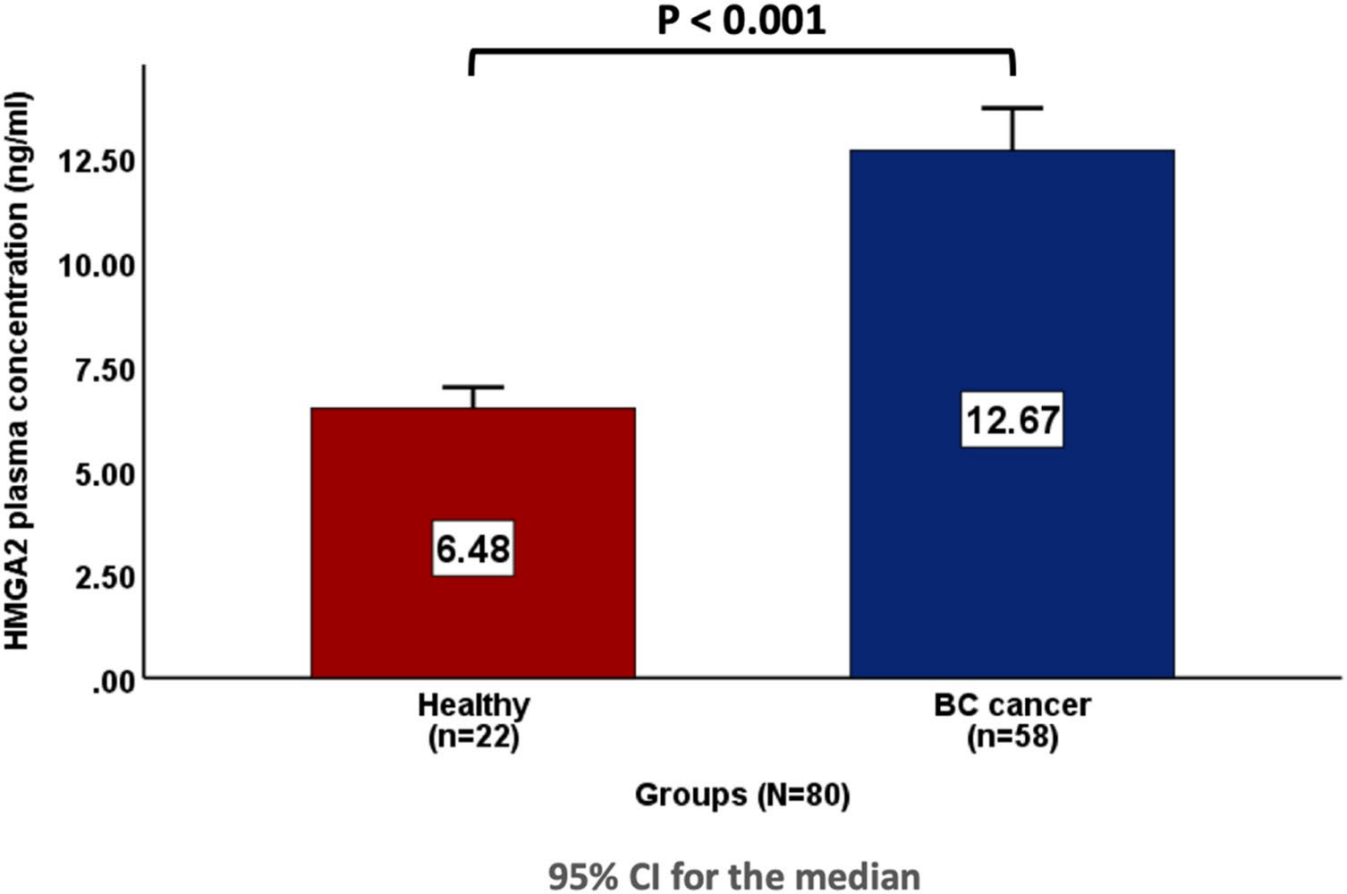
Comparison of plasma HMGA2 levels between the BC group and healthy group

This study indicated that plasma HMGA2 levels were higher in the BC group (12.67 [10.28–15.57] ng/ml) than in the healthy group (6.48 [4.95–7.01] ng/ml) when applying Mann–Whitney test (*P* < 0.001), as shown in Fig. 1.

### Comparison of plasma HMGA2 levels in BC patients according to the clinicopathological features

**Fig. 2:**
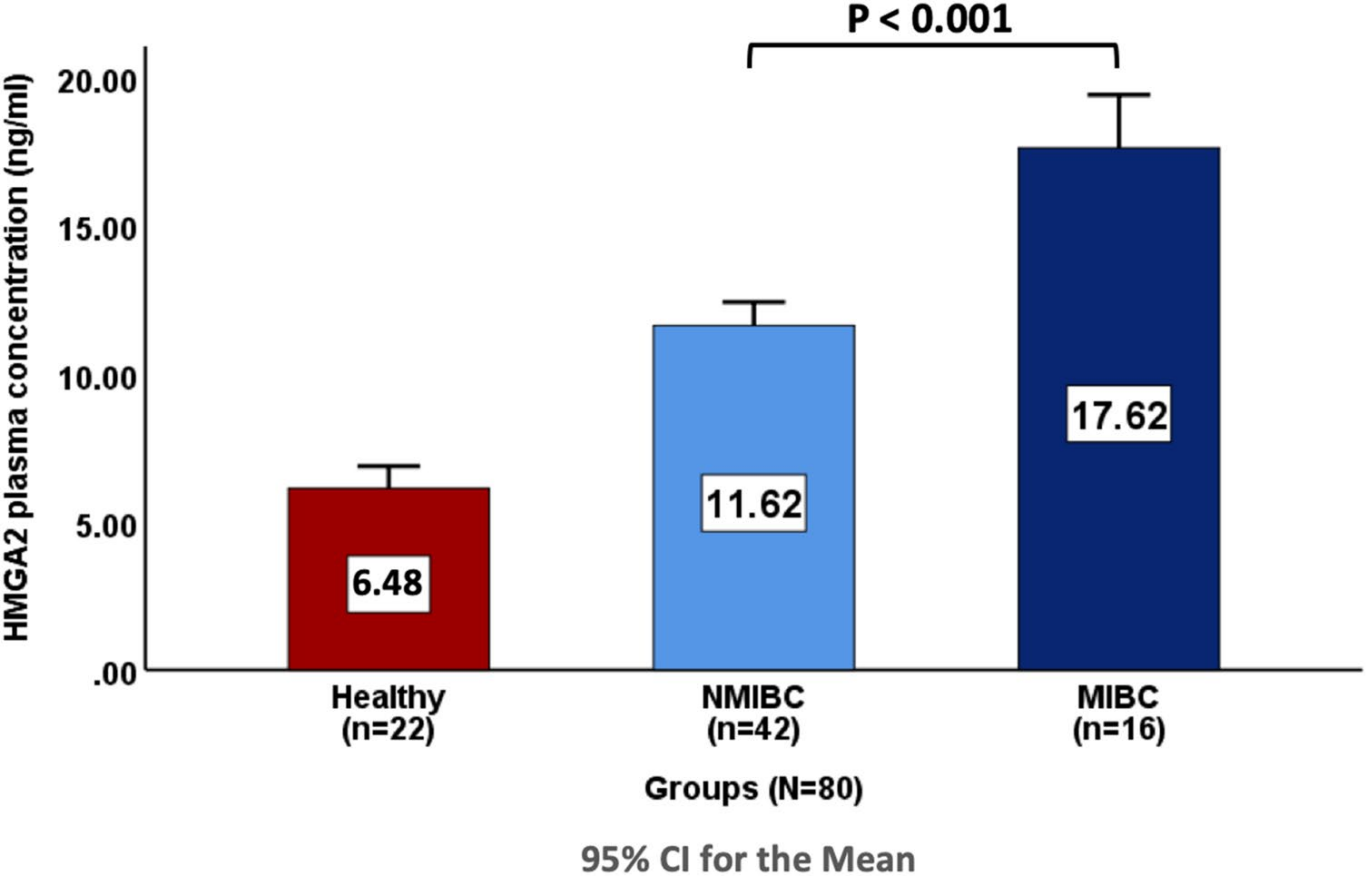
Comparison of plasma HMGA2 levels in BC patients according to clinicopathological features

The patients were classified according to tumor stage and EAU classification, and the association between each classification and plasma HMGA2 levels was investigated. This study found a significant difference in plasma HMGA2 level between NMIBC (pTa-pT1) and MIBC (pT2-pT3) when applying t-test, as HMGA2 levels were higher in MIBC (pT2-pT3) (17.62 ± 3.37 ng/ml) than in NMIBC (pTa-pT1) (11.62 ± 2.56 ng/ml) (*P* < 0.001), as shown in Fig. 2.

No significant differences were found between pTa and pT1 (*P* value = 1) or between pT2 and pT3 (*P* value = 0.117), and also between individuals within the groups according to gender (Table 1).

### Comparison of plasma HMGA2 levels between high grade BC patients and low grade BC patients

**Fig. 3:**
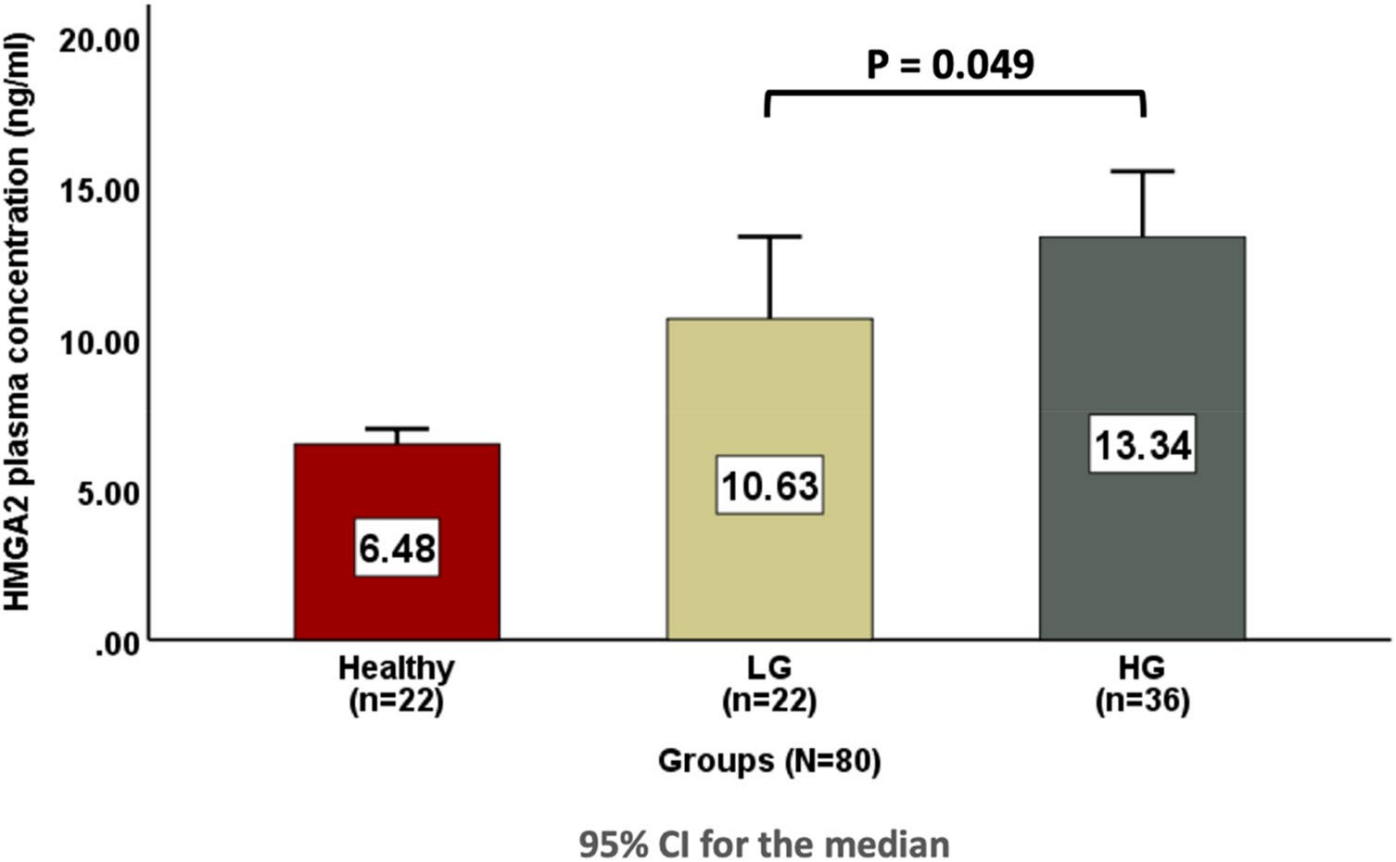
Comparison of plasma HMGA2 levels between high grade BC patients and low grade BC patients

In the BC group (*n* = 58), 22 patients (37.93%) were classified as low grade, and 36 patients (62.07%) were classified as high grade. A significant difference in the plasma HMGA2 level was found between low grade BC patients and high grade BC patients when applying Mann–Whitney test, as the plasma HMGA2 level was higher in high grade BC patients (13.34 [11.7–17.28] ng/ml) than in low grade BC patients (10.63 [8.6–13.68] ng/ml), with a *P*-value = 0.049, as shown in Fig. 3.

### ROC curve of plasma HMGA2 levels to determine the cut-off value that discriminates between bladder cancer and healthy group

**Fig. 4:**
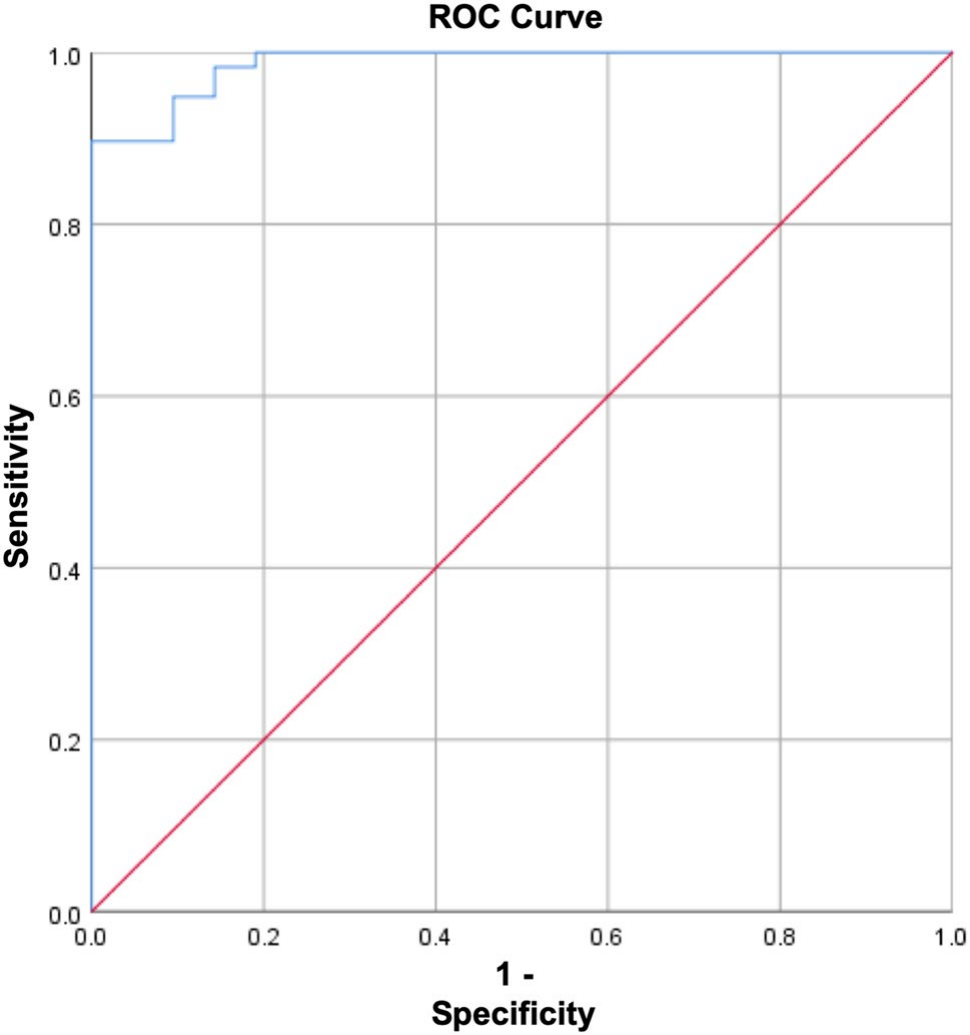
ROC curve of plasma HMGA2 levels to determine the cut-off value that discriminates between bladder cancer and healthy group

Based on the presence of a statistically significant difference in plasma levels of HMGA2 protein between the bladder cancer patients group and the healthy group when applying Mann–Whitney test, a receiver operating characteristic (ROC) curve was constructed to study the sensitivity and specificity of plasma HMGA2 levels in discriminating the bladder cancer group and healthy group (shown in Fig. 4). Various cut-off values of HMGA2 protein level in the plasma were obtained, and the area under the curve was calculated (Table 2). 8.41 ng/ml was considered as the cut-off value, where the sensitivity and specificity were 94.8% and 90.5%, respectively.

**Table 2:** Area under the curve description

Area under the curve	Standard error	*P*-value*
0.987	0.009	** < 0.0001**

*P-value > 0.05 is not significant

## Discussion

Bladder cancer diagnosis and follow-up procedures are painful, invasive, and represent a financial burden for patients, and the non-invasive, easy-to-analyze biomarkers capable of diagnosing bladder cancer at early stages and managing it have not yet been approved. Extensive research has demonstrated the involvement of HMGA2 protein in carcinogenesis at multiple levels. It intervenes in crucial processes such as cell cycle regulation, apoptosis, angiogenesis, epithelial-to-mesenchymal transition, cancer cell stemness, and DNA damage repair mechanisms, ultimately promoting cancer cell survival. The HMGA2 protein is an oncofetal protein whose expression increases during embryogenesis but decreases or is absent in adult tissues to increase again during carcinogenesis (Rogalla et al. 1996).

In this context, many histological studies were conducted to investigate the relationship between HMGA2 protein expression levels and histological and clinical characteristics of bladder cancer tissues, and compared them with normal adjacent tissues. Yang et al. (China, 2011) (Yang et al. 2011) conducted one of these studies, which included 148 tissue samples in which HMGA2 protein levels were studied by ImmunoHistoChemical staining (IHC), and 44 tissue samples in which HMGA2 mRNA levels were studied by quantitative reverse-transcription Polymerase Chain Reaction qrt-PCR. Also Ding et al. (China, 2014) (Ding et al. 2014) studied HMGA2 protein levels in 49 bladder cancer tissue samples using IHC. Both studies indicated that that the expression of HMGA2 was increased in BC tissues compared with adjacent normal tissues, and the higher levels of HMGA2 protein expression were associated with advanced cancer grade and stage. While in other study conducted by Krafft et al. (Germany, 2019) (Krafft et al. 2019), which included 106 tissue samples from patients with bladder cancer, 49 of which were from patients who had not yet undergone any chemotherapy, and were studied using (IHC), it was found that increased tissue levels of HMGA2 protein were associated with increased resistance to cisplatin in bladder cancer. Increased tissue levels were also associated with the presence of metastases. The study also revealed that increased HMGA2 protein staining was associated with poor overall survival and shorter progression-free survival in the subgroup of patients who had received at least two courses of chemotherapy, which demonstrates the importance of HMGA2 protein tissue levels in evaluating the prognosis of bladder cancer. Therefore, this study aimed to investigate the presence and levels of HMGA2 protein in the plasma of bladder cancer patients and its association with stage, grade, and muscle invasion.

This study confirmed the presence of the HMGA2 protein in the plasma. Despite that HMGA2 is a nuclear protein that does not contain Signal Peptide (SP) (Keller et al. 2008), the truncated form of HMGA2 protein—associated with tumor aggressiveness—is present in the cytoplasm in addition to the nucleus (Campbell et al. 2023), as well as the phosphorylation occurring on both sides of the Nuclear Localization Signal (NLS) presented in the second AT-hook may facilitate the change of the HMGA2 localization from the nucleus to the cytoplasm, which facilitate its secretion by an independent-endoplasmic reticulum/Golgi pathway (Cattaruzzi et al. 2007). The extracellular vesicle-based secretion pathway mediated by caspase1 during pyroptosis (a form of regulated cell death) has been considered as one of the proposed pathways in which inflammasome sensors, after recognizing damage-associated molecular patterns (DAMPs), bind to receptors that activate caspase 1, which in turn cleaves Gasdermin D (GSDMD) and induces pyroptosis. This process leads to the release of HMGA2 via an extracellular vesicle-based caspase1-mediated pathway (Chang et al. 2015). Increased expression of caspase 1 was observed in bladder cancer cells, as well as the prevalence of pyroptosis (Wang et al. 2023), which suggested the secretion of HMGA2 in the plasma of bladder cancer patients. This study also showed higher plasma levels of the HMGA2 protein in the BC group than in the healthy group. In cancer, the epigenetic mechanisms regulating the expression of the HMGA2 protein are lost because of deletions or mutations in the 3′ UnTranslated Region (UTR) of HMGA2 mRNA associated with cancer, which contains MiRNA Response Elements (MREs), as miRNA-mediated regulation is the main epigenetic mechanism that controls HMGA2 expression, and this leads to a significant increase in HMGA2 levels in cancer (Ergun and Oztuzcu 2015; Lee and Dutta 2007; Mayr et al. 2007). This study also indicated that plasma HMGA2 protein levels were higher in (pT2-pT3) patients, which are MIBC, than in (pTa-pT1) patients, which are NMIBC. Studies have shown that the HMGA2 protein stimulates carcinogenesis by activating E2F1 and AP-1 and increasing Cyclin A2 expression in the cell cycle, in addition to preventing the expression of p53 and stimulating its degradation (Martino et al. 2019; Mansoori et al. 2021b). the HMGA2 protein also stimulates angiogenesis by increasing the expression of essential growth factors, especially VEGF-A, VEGF-C, FGF-2, TGF-β, and IGF2BP2 (Sakata et al. 2019). During cancer development, the HMGA2 protein participates in the EMT process, leading cancer cells to migrate and invade by increasing the expression of mesenchymal markers such as vimentin, Snail1, and Twist and reducing the expression of epithelial markers such as E-cadherin and Occludin (Li et al. 1997; Shi et al. 2016), as well as stimulating extracellular matrix degeneration to facilitate cancer cell migration and invasion through increasing the expression of MMP2 and MMP9 (Yan et al. 2021). In addition, the HMGA2 protein also stimulates the TGF-β, MAPK, and PI3K signaling pathways, which in turn increases HMGA2 expression within a positive feedback loop, causing a significant increase in HMGA2 levels as the tumor reaches the stage of muscle invasion (Tan et al. 2016; Voon et al. 2013; Ayoubi et al. 1999).

We also found a correlation between plasma HMGA2 level and BC grade, which is essentially related to the differentiation status of cancer cells; the higher the grade is, the less common the differentiation and the poorer the prognosis. This study demonstrated a higher plasma HMGA2 levels in high grade BC patients than in low grade BC patients, as studies have shown an important role for the HMGA2 protein in promoting the expression of cancer stem cell markers, especially CD44, Oct4, c-Myc, ALDH1, and Twist1 (Mansoori et al. 2020, 2021a; Sun et al. 2017), additionally to the activation of the Wnt/β-catenin pathway, which is known to be responsible for self-renewal, further promoting cancer cell aggressiveness, metastasis, and resistance to cancer therapies (Zha et al. 2013).

Accordingly, plasma HMGA2 protein levels can be used as a potential non-invasive marker for managing bladder cancer.

This study has many limitations due to limited self-funding; the first limitation is the relatively limited number of participants, and the second limitation is the lack of supporting in vitro or histological study. Also, in this study the healthy control group comprised individuals without hematuria. Although participants with hematuria would have been the ideal control group, they were excluded due to not obtaining cases with hematuria that were not attributed to inflammatory or necrotic causes.

## Conclusion

In this study, we confirmed that the plasma HMGA2 protein level elevated in patients with bladder cancer and that the plasma HMGA2 protein level was higher in patients with MIBC than in patients with NMIBC. Our study also investigated plasma HMGA2 levels in patients with different BC grades and confirmed that the plasma HMGA2 levels were higher in patients with high grade BC than in patients with low grade BC. Thus, the plasma HMGA2 concentration represents a potential non-invasive marker for bladder cancer diagnosis and management.

## Data Availability

All materials and all data generated during this study are included in this article.

## Abbreviations

ALDH1: Aldehyde dehydrogenase 1
AP-1: Activator protein-1
BC: Bladder cancer
BMREC: Biomedical Research Ethics Board
c-Myc: Cellular myelocytomatosis
CD44: Cluster of differentiation 44
DAMPs: Damage-associated molecular patterns
DNA: Deoxyribonucleic acid
E2F1: E2 promoter binding factor 1
EAU: European Association of Urology
ELISA: Enzyme-linked immunosorbent assay
EMT: Epithelial–mesenchymal transition
FGF: Fibroblast growth factor
GLOBOCAN: Global observatory of cancer
GSDMD: Gasdermin D
HMG: High mobility group
IGF2BP2: Insulin-like growth factor 2 mRNA-binding protein 2
IHC: Immunohistochemical staining
IQR: Interquartile rang
MAPK: Mitogen-activated protein kinase
MIBC: Muscle-invasive bladder cancer
miRNA: MicroRNA
MMP: Matrix metalloproteinases
MRE: MicroRNA response element
mRNA: Messenger RNA
NLS: Nuclear localization signal
NMIBC: Non-invasive bladder cancer
Oct4: Octamer-binding transcription factor 4
PI3K: PhosphatidylInositide 3-kinase
qrt-PCR: Quantitative reverse-transcription polymerase chain reaction
ROC: Receiver operating characteristic
SP: Signal peptide
Std: Standard deviation
TGF-β: Transforming growth factor beta
TURBT: Transurethral resection of bladder tumor
UTR: Untranslated region
VEGF: Vascular endothelial growth factor
Wnt: Wingless-related integration site
